# Numerical Simulation and Experimental Validation of Squeeze Casting of AlSi9Mg Aluminum Alloy Component with a Large Size

**DOI:** 10.3390/ma15124334

**Published:** 2022-06-19

**Authors:** Jufu Jiang, Jing Yan, Yingze Liu, Guoquan Hu, Ying Wang, Changjie Ding, Dechao Zou

**Affiliations:** 1 School of Materials Science and Engineering, Harbin Institute of Technology, Harbin 150001, China; yannjing@foxmail.com (J.Y.); liuyingze1995@foxmail.com (Y.L.); 13840355231@163.com (G.H.); 2 School of Mechatronics Engineering, Harbin Institute of Technology, Harbin 150001, China; 3 Dalian Innovation Die-Casting Co., Ltd., Dalian 116600, China; dhmdcj@163.com (C.D.); zoudechao@innovation-dalian.com (D.Z.)

**Keywords:** squeeze casting, numerical simulation, temperature field, stress field, defects prediction

## Abstract

The squeeze casting process for an AlSi9Mg aluminum alloy flywheel housing component was numerically simulated using the ProCAST software, and orthogonal simulation tests were designed according to the L16 (4) ^5^ orthogonal test table to investigate the alloy melt flow rule under four factors and four levels each of the pouring temperature, mold temperature, pressure holding time and specific pressure, as well as the distributions of the temperature fields, stress fields and defects. The results showed that the flywheel housing castings in all 16 test groups were fully filled, and the thinner regions solidified more quickly than the thicker regions. Hot spots were predicted at the mounting ports and the convex platform, which could be relieved by adding a local loading device. Due to the different constraints on the cylinder surface and the lower end surface, the solidification was inconsistent, the equivalent stress at the corner junction was larger, and the castings with longer pressure holding time and lower mold temperature had larger average equivalent stress. Shrinkage cavities were mainly predicted at mounting ports, the cylindrical convex platform, the peripheral overflow groove and the corner junctions, and there was also a small defect region at the edge of the upper end face in some test groups.

## 1. Introduction

With the rapid development of the world economy, the impact of global warming and the energy crisis are becoming increasingly serious [1], and environmental protection and energy conservation have gradually become themes of scientific research. Since the 1980s, a wave of lightweight technology has emerged worldwide, involving many fields such as automotive engineering, aerospace and weapons [2]. Due to the characteristics of high specific strength, good corrosion resistance, weldability [3] and especially secondary recycling [4], aluminum alloys have attracted a great deal of attention from researchers. Compared with other advanced materials such as ultra-high-strength steel, these alloys can achieve excellent weight reduction and better fuel economy [5]. Studies show that if aluminum alloy was used to replace steel, the mass of an automobile engine could be reduced by nearly 30%, the mass of a wheel could be reduced by nearly 50% and the total weight of an automobile could be reduced by 30~40% [6,7], showing a considerable benefit.

Aluminum alloys have a good processing performance, and they are suitable for a variety of manufacturing processes. Both wrought and cast aluminum alloy can be used to manufacture various automobile parts [8]. The wrought aluminum alloy has good relative density and mechanical properties, but its high production cost and low production efficiency limit its wide application [9,10]. In contrast, cast aluminum alloy is more popular due to its low production cost, but the performance of an aluminum alloy produced by the casting process is not so desirable [11]. Squeeze casting technology, precisely combining the advantages of both casting and forging, has broad application prospects in terms of low cost, a short process and high-quality mass production, and is a key technology for the future replacement of steel with aluminum, using casting instead of forging to achieve high performance and lightweight manufacturing of large complex structural parts [12,13].

Squeeze casting, also known as liquid die forging, refers to the combined casting–forging process of filling, solidification and forming of liquid or semisolid metal under pressure by injecting a certain amount of liquid metal into the mold cavity and applying high mechanical pressure [14,15]. The most prominent feature of squeeze casting is the fact that the high pressure in the manufacturing process can form components with complex shapes and dense microstructures [16]. Furthermore, high pressure can lead to a decrease in grain sizes and casting defects such as shrinkage cavities and porosity. As a result, the castings have better mechanical properties [17,18]. Squeeze casting can involve direct squeeze casting or indirect squeeze casting. Since the gating system is not used in direct squeeze casting, the metal utilization rate can reach up to 95% [19]. Compared with direct squeeze casting, indirect squeeze casting has more advantages in the production of thin-walled and complex parts. In addition, indirect squeeze casting can produce complex near-net-shape castings with high surface roughness. It can not only reduce the subsequent manufacturing processing of parts, saving energy and capital, but also shorten the production cycle. Against the background of energy shortages, manufacturing industries around the world are seeking high-performance and energy-saving technologies. Indirect squeeze casting has great development potential for future casting production.

At present, the characteristics of indirect squeeze casting mean that that it is mainly used for the forming of small castings. With the improvement of the process, indirect squeeze casting is developing towards being used for large and complex castings. Indirect squeeze casting technology for large and complex parts has gradually become an important technology in the casting field [20]. The quality of squeeze-cast parts is affected by many process parameters, and the full potential of the process can only be realized when production is carried out with optimized process parameters. Numerical simulation is a good method of truly reflecting the filling and solidification process, deeply analyzing the distribution of the flow field, temperature field and stress field in the forming process and predicting possible defects, in order to seek better solutions [21], and it plays a significant role in optimizing the process design and mold structure, improving the quality of castings, shortening the trial production cycle, reducing production costs and improving the economic benefits of production.

In view of the above background, numerical simulation research on squeeze casting technology for aluminum alloy engine flywheel housing components for large vehicles was proposed in this study. The engine flywheel housing component is usually installed between the gearbox and the engine, in order to connect them. The component belongs to a complex and large-sized shell-structure component with large wall-thickness differences, which is complicated in shape and difficult to form, and this has always been a difficulty in the casting field. Some researchers have studied casting–forming technology for thin-walled shell structures and the numerical simulation of squeeze casting. G. Hébert et al. [22] compared the bending fatigue strength and tensile properties of A363 aluminum alloy thin-walled components formed by die casting, vacuum die casting, squeeze casting and gravity casting, and the results showed that the mechanical properties of thin-walled components formed by squeeze casting were the best. R. Pastircák et al. [23] studied the variation in strength and plasticity of thin-walled components with different thicknesses under squeeze casting and gravity casting, and found that the strength of the squeeze-cast component was increased by 37% compared with that of the gravity-cast component with the same thickness of 8 mm. In addition, thinner gravity-cast components had greater strength and plasticity, while the change rule for squeeze-cast components was the opposite. This indicated that it was still difficult to form a component with the required performance by squeeze casting. Du et al. [24] studied the effect of pouring temperature on the properties of a squeeze-cast 2A14 aluminum alloy thin-walled shell. The results showed that a higher pouring temperature was more conducive to the completion of filling (720~740 °C was the optimal filling temperature range), giving formed thin shell parts with a fine grain size and no dendrites. Ying et al. [25] studied the influence of filling velocity on the forming quality of thin-walled parts in the process of squeeze casting, and obtained an optimal filling velocity theoretically, which was of significance for guiding the design of filling speeds for thin-walled parts in the squeeze casting process. However, there was a lack of experiments to verify whether the filling velocity obtained by the theory could reduce the generation of defects such as air entrapment and cold shuts in thin-walled parts in practice. Xu et al. [26] used the ProCAST software to simulate the filling speed for indirect squeeze casting. Through the simulation analysis of the filling of a type of panel, it was found that the filling effect was best when the velocity v was equal to the filling time t, providing better process parameters for similar squeeze castings. Li et al. [27] simulated the squeeze casting process for an automobile control arm using MAGMASOFT software and overcame the occurrence of shrinkage defects by local pressure supplementing and cooling-line setting. Tang et al. [28] proposed a thermal–mechanical coupling simulation method for the solidification process for squeeze casting based on ANSYS, according to the basic physical laws and characteristics of the solidification process, thus describing the temperature change in the casting solidification process.

However, there is a lack of accurate prediction methods and effective methods of controlling defects in the forming process of complex components with large wall-thickness differences in the casting field. In order to investigate the solidification sequence of the flywheel shell forming process and predict the location of the defects, this study compared and analyzed the distributions of the temperature fields, stress fields and defects for flywheel housing components formed by squeeze casting under different process conditions through numerical simulation, providing theoretical guidance for the adoption of local loading and forced feeding. The prediction of casting defects was one of the main purposes. This is a key part of casting solidification process simulation which can provide an important theoretical basis for subsequent practical production and promote the application of the squeeze casting and forming process in the manufacturing of load-bearing structural parts with excellent performance.

## 2. Numerical Simulation Experiment Scheme and Preprocessing

**Figure 1:**
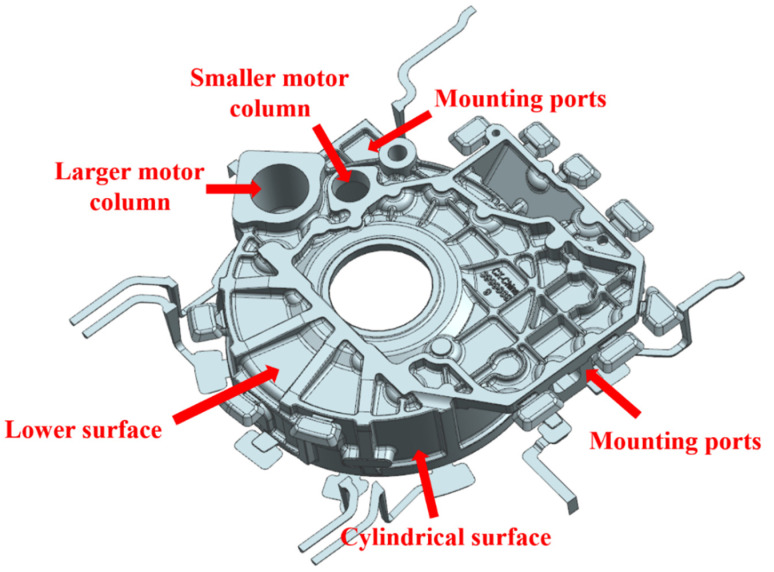
Diagram of AlSi9Mg aluminum alloy flywheel housing component.

The flywheel housing component is used to connect the engine and the gearbox through the shafts through the two motor columns. Figure 1 shows a diagram of the flywheel housing casting. AlSi9Mg aluminum alloy was selected as the material. The chemical composition is shown in Table 1. The ProCAST software was used to simulate the squeeze casting process for flywheel housing components, and the flow rule for molten metal in the filling process was investigated. Considering the influence of various process parameters on the comprehensive performance of castings, this paper mainly focused on the distributions of the temperature fields, stress fields and defects in the flywheel housing components under different pouring temperatures, mold temperatures, pressure holding times and specific pressures.

### 2.1. Experimental Parameters Determination

The pouring temperature directly affects the solidification time and quality of castings, and it also has a great impact on the service life of the mold [29]. A high pouring temperature will lead to an increase in air absorption by the aluminum melt, resulting in porosity and other defects, and it will also cause mold wear and reduce the service life of the mold. However, a low pouring temperature will make the molten metal solidify early in the filling process, resulting in defects such as insufficient filling and cold shuts [30]. Therefore, the selection of pouring temperature is crucial to the casting performance. According to the thermal physical properties and actual production rules of AlSi9Mg aluminum alloy, four different temperature levels of 645 °C, 650 °C, 655 °C and 660 °C were selected for the simulation in this experiment.

The mold temperature also affects the filling process of the aluminum alloy. A high mold temperature reduces the solidification speed of the molten metal, resulting in a coarse microstructure in the formed castings and increasing the tendency to form shrinkage cavities and porosity. In addition, a higher mold temperature leads to easier deformation under pressure, resulting in mold wear and reducing the service life of the mold. However, a low mold temperature leads to a rapid decline in the temperature of the molten metal and a deterioration in the fluidity, which leads to insufficient filling and cold shuts [31]. After analyzing and summarizing the change rules of the actual mold temperature of the flywheel housing squeeze casting, four different mold preheating temperature levels were explored in this study, with each level set as shown in Table 2. The temperature settings of the front side mold and rear side mold were consistent with the setting for the left side mold.

The selection of pressure holding time depends on the shape and size of the casting, the mold temperature and the pouring temperature. A long pressure holding time makes the casting tighten on the punch pin and affects the demolding, and it also reduces the service life of the mold. A short holding time may lead to incomplete solidification of the castings. According to industrial production experience, four pressure holding time levels of 20 s, 25 s, 30 s and 35 s were used in this study.

The specific pressure also has an important influence on the forming process. Generally, the higher specific pressure acting on the casting, the finer microstructure and the better the mechanical properties of the components [32]. However, excessive pressure will lead to a leakage of molten metal, which has an impact on production safety. The specific pressure refers to the ratio of the force exerted by the lower injection cylinder to its action area on the casting. According to an actual measurement, the action area on the casting of the lower injection cylinder was about 35,298.94 mm^2^. Considering the situation, four specific pressure levels were used, as shown in Table 3.

### 2.2. Numerical Simulation Experiment Scheme

The study mainly revealed the influence of pouring temperature, mold temperature, pressure holding time and specific pressure on the squeeze casting process for a flywheel housing component, and each factor was designed with four level variations, as shown in Table 4. For a comprehensive test, 256 groups of tests would be required, and the workload would be huge. Therefore, it was decided to adopt the four factors and four levels of an orthogonal test to replace all tests with partial tests, and the L16 (4) ^5^ orthogonal test table was chosen [33] so that only 16 groups of tests were needed, which greatly reduced the workload. The factors and levels of the orthogonal test are shown in Table 5.

### 2.3. Model Establishment

**Figure 2:**
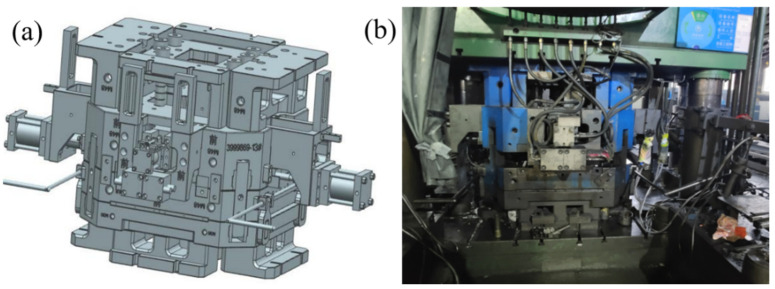
Mold for engine flywheel housing component: (**a**) three-dimensional model; (**b**) photograph.

**Figure 3:**
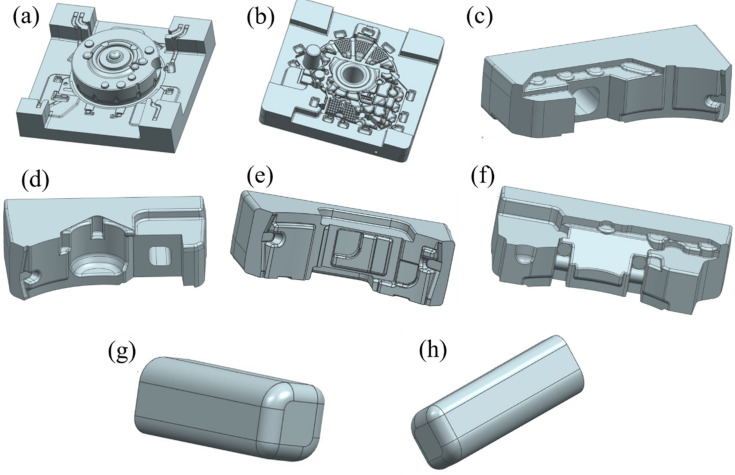
Simplified 3D mold drawing of engine flywheel housing: (**a**) upper die; (**b**) lower die; (**c**) left side die; (**d**) right side die; (**e**) rear side die; (**f**) front side die; (**g**) left pressure compensation bar; (**h**) right pressure compensation bar.

A three-dimensional model and photograph of the mold used in the study are shown in Figure 2. The cavity was mainly composed of a lower mold, an upper mold, four side molds and two local pressure compensation devices (i.e., local loading devices for reducing the defects in hot spots). In order to reduce the difficulty and time of the numerical simulations, UG software was used to simplify the mold before simulation. Some parts of the mold not needed for the simulation were removed, and only the cavity parts of the mold were retained, including the upper die, lower die, front side die, rear side die, left side die, right side die, left pressure compensation bar and right pressure compensation bar, as shown in Figure 3.

### 2.4. Meshing

**Figure 4:**
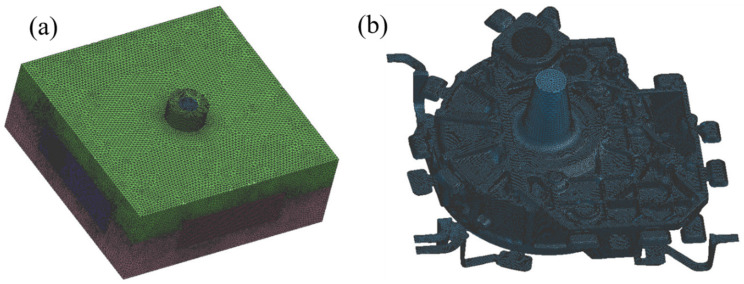
Diagrams of the mold and casting after mesh division: (**a**) mold; (**b**) casting.

The simplified models were imported into the ProCAST software, and the Visual-Mesh module was used to check and repair the 3D model, assemble the different parts of the mold cavity, conduct the body cross-check and conduct the surface mesh division, surface mesh inspection, body mesh division and body mesh inspection in turn. Considering the calculation time and accuracy, the distance between points on the outer contour lines of the upper die and lower die away from the casting was set to 10 mm, and the distance between points on the outer contour of each side die away from the casting end was set to 6 mm. Due to the complex shape with many complicated rounded corners in the casting and the necessity of considering the subsequent analysis of the stress field, temperature field and defects of the casting, the distance between points on each contour line of the casting surface was set to 3 mm. In the process of meshing, it was important to avoid unqualified meshing, and therefore the differences between the distances between points on adjacent lines could not be too large. Figure 4 shows a diagram of the mold and casting after mesh division. The surface mesh number was 467732, and the body mesh number was 10262798.

### 2.5. Set of Initial Conditions

The meshed three-dimensional model was successively set in terms of gravity direction, material, initial temperature of material, heat transfer coefficient, gate, pouring time, boundary conditions, cooling conditions, simulation time, etc.

According to the model and the actual situation, the gravity direction was set as downward, perpendicular to the upper surface of the upper die, and the gravity acceleration was set at 9.8 m/s^2^. The mold material was set as H13 steel, and the casting material was set as AlSi9Mg aluminum alloy. According to the actual motion stroke and speed of the lower injection cylinder of the equipment, the pouring time was set to 5 s. The mold was set to contact with air of 20 °C, and the heat transfer coefficient between the two was set to 10 W·m^−2^·°C^−1^. The heat transfer coefficient between the casting and the mold was mainly affected by the specific pressure, with higher specific pressures leading to higher heat transfer coefficients [34,35]. The relationship is shown in Equation (1) [36], and the calculated heat transfer coefficients under different specific pressures were as shown in Table 6.

(1) h=1990.5+94.8P,

where *h* is the heat transfer coefficient between the casting and the mold, and *P* is the specific pressure.

## 3. Results and Discussion

### 3.1. Analysis of Filling Process

**Figure 5:**
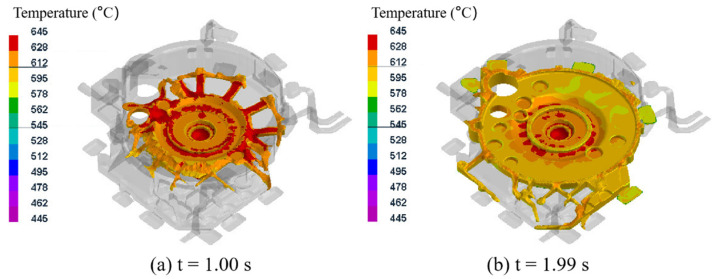

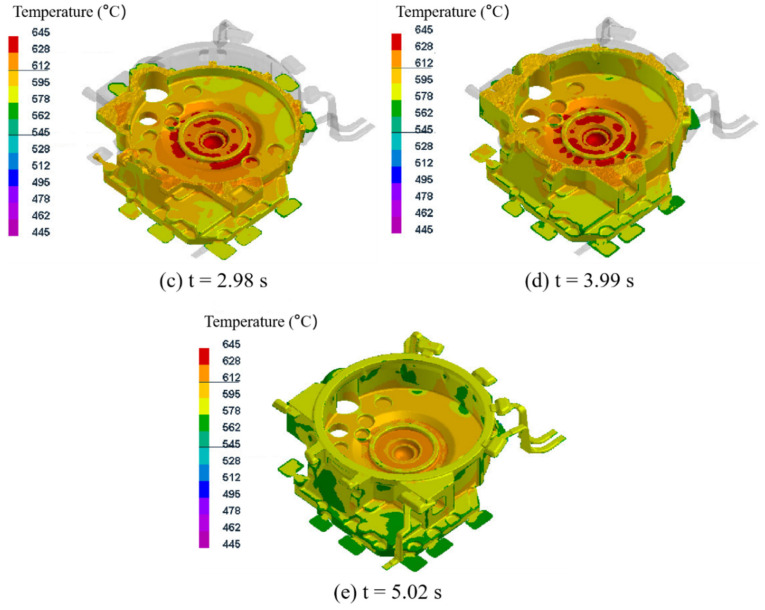
Temperature field distribution during mold filling process for test group No. 1.

Figure 5 shows the temperature field distribution during the mold filling process for test group No. 1 (pouring temperature of 645 °C, mold temperature at level A, pressure holding time of 20 s, specific pressure of 17.00 MPa). The aluminum melt entered the mold cavity from the gate in the anti-gravity direction and began to fill from the center of the flywheel housing component. The aluminum alloy liquid first flowed into the reinforcement rib on the lower surface of the flywheel housing component, and then filled the whole cavity in 5 s from bottom to top, with no insufficiently filled region. During the whole filling process, the flow velocity of the aluminum liquid was stable, and there was no liquid splash leading to air entrapment.

It can be seen that after 1.00 s of filling, the aluminum melt filled the sprue and began to spread from the center to the surrounding areas along the thicker rib on the lower surface of the flywheel housing component (Figure 5a). After filling for 1.99 s, the entire lower end face of the flywheel housing was almost full, and the temperature at the peripheral overflow groove was lower than that at other positions (Figure 5b), because the temperature here decreased rapidly due to the thin peripheral overflow groove [37]. After filling for 2.98 s, the mold filling rate reached 61.9%, and it could be seen that the temperature of the direct contact surface between the aluminum alloy liquid and the die was lower, while the temperature of the non-contact surface was higher (Figure 5c). After filling for 3.99 s, the temperature at the various corners and edges of the flywheel housing was relatively low, dropping to between 552 °C and 578 °C (Figure 5d). After filling for 5.02 s, the filling process was complete for the entire flywheel housing, and the filling rate reached 100% (Figure 5e). At this time, the solid fraction was 6.9%, and the average temperature of the aluminum alloy liquid was 606.4 °C, indicating that the flywheel housing was fully filled and that the aluminum melt still had good fluidity after filling, which made it possible to exert a certain pressure for forced feeding through the lower injection cylinder during the solidification process [27]. The simulation experiments were carried out for 16 groups, and the results showed that the filling process was roughly the same under each process parameter and that all the aluminum alloy flywheel housing castings were completely filled, with no filling dissatisfaction.

### 3.2. Temperature Field Analysis of Castings and Mold

**Figure 6:**
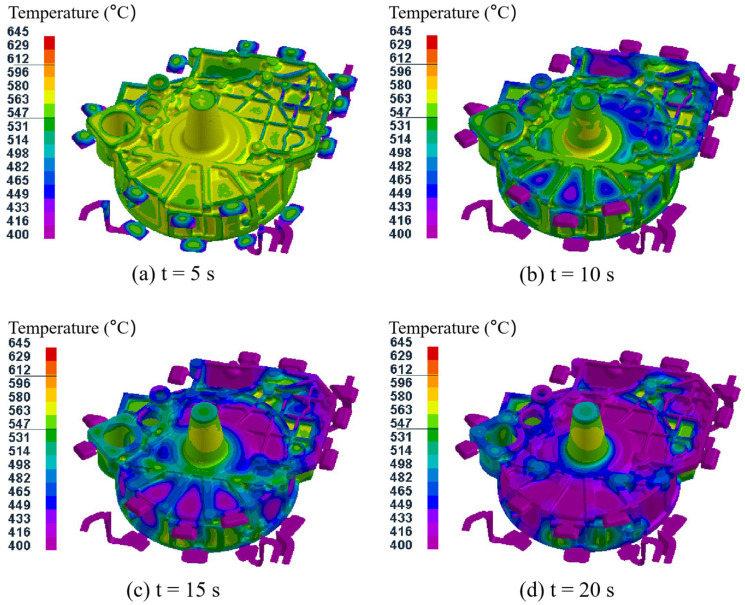
Temperature field distribution during casting solidification process for test group No. 1.

Figure 6 shows the temperature field distribution during the solidification process of the castings in test group No. 1 (pouring temperature of 645 °C, mold temperature at level A, pressure holding time of 20 s, specific pressure of 17.00 MPa). As shown in Figure 6a, when the pressure holding time reached 5 s, cooling first occurred at the peripheral overflow groove, and the average temperature of the casting was 572 °C. After 10 s, the surface of the flywheel housing component in contact with the mold and the region with thinner wall thickness also cooled down gradually (Figure 6b). At this time, the average temperature of the casting was 540 °C, which is below the solidus temperature of 545 °C for the AlSi9Mg aluminum alloy. When the pressure holding time reached 15 s, the solidification area was further expanded from the thin-walled region, and the average temperature of the casting was further reduced to 510 °C (Figure 6c). When the pressure holding time reached 20 s, the temperature of the whole flywheel housing casting had reduced to below the solidus temperature of the alloy (Figure 6d). At this time, the average temperature of the casting was 480 °C, and the pressure holding process was complete. As shown in Figure 6d, after the pressure holding process, the temperature at the larger motor column, left and right mounting ports and cylindrical convex platform was higher than that in other regions. This is because the thickness of these regions was relatively large, resulting in a low cooling speed [38].

Therefore, on the lower surface of the flywheel housing, the solidification process started from the edge area away from the gate and then gradually expanded to the center, and it can be speculated that shrinkage cavities will be distributed close to the gate position. At the same time, due to the pressure holding effect for forced feeding by the lower injection cylinder, the shrinkage cavity around the gate could be effectively controlled [39,40].

**Figure 7:**
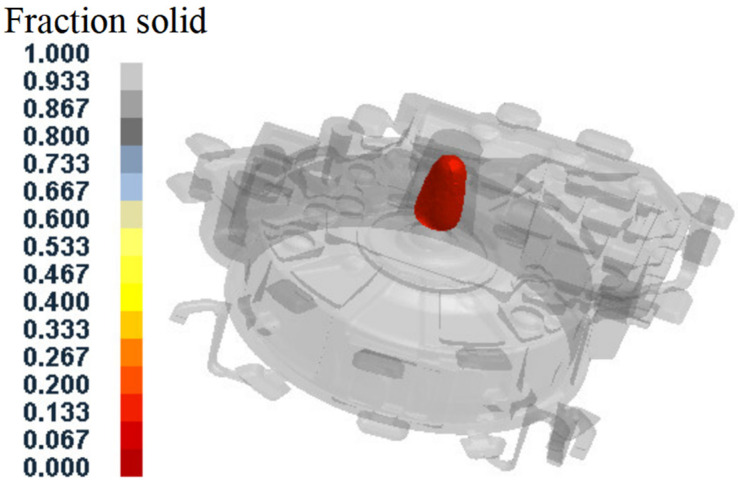
Position of material with solid fraction below 20% after pressure holding process.

**Figure 8:**
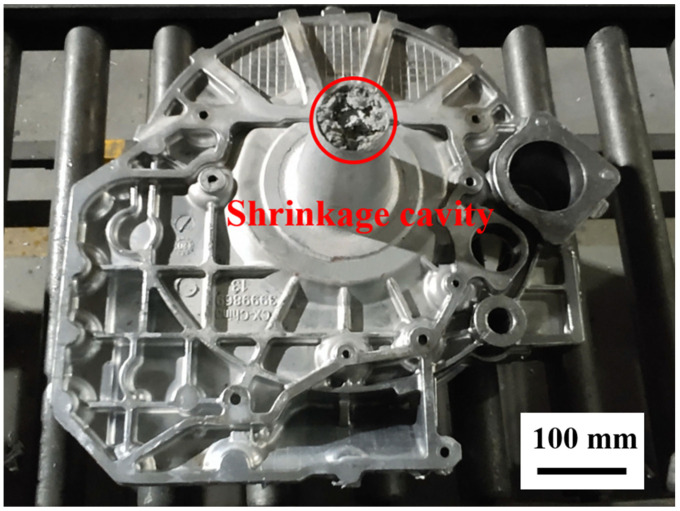
Produced flywheel housing component with sprue.

Figure 7 shows the distribution of the regions with a solid fraction below 20% after the pressure holding process. It can be seen that the alloy at the sprue position was not completely solidified, and therefore it would continue cooling naturally until complete solidification after the pressure holding process. Figure 8 shows the produced flywheel housing component with the sprue. It illustrates that the shrinkage cavity was concentrated at the sprue position, due to the final solidification. However, in actual production, the solidified metal at the sprue position is not used and can be removed from the component body. Therefore, shrinkage cavities concentrated at the sprue position will not affect the forming quality of the squeeze-cast components.

**Figure 9:**
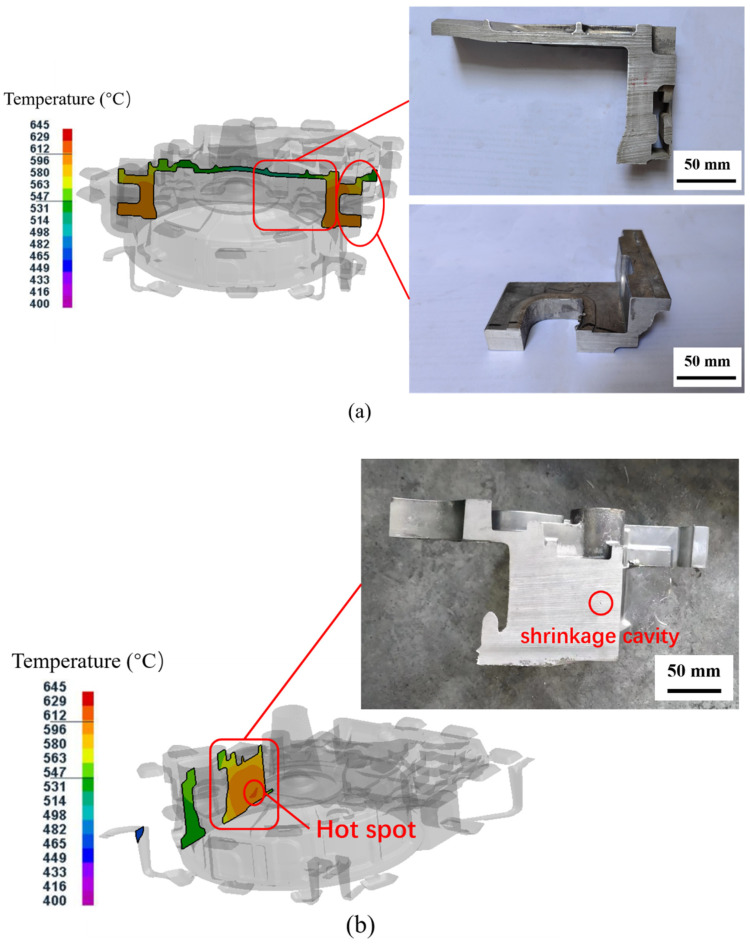
Temperature field distribution and photographs of the cross section at the mounting ports for test group No. 16: (**a**) right mounting port; (**b**) left mounting port.

Figure 9 shows the temperature field distribution and the photographs of the cross section at the mounting ports with a local loading device, for test group No. 16 (pouring temperature of 660 °C, mold temperature at level D, pressure holding time of 30 s, specific pressure of 22.66 MPa) after the pressure holding process. Due to the large wall thickness of the mounting ports, it was easy to produce hot spots, and such areas were prone to form defects such as shrinkage cavities and porosity [41,42]. However, it can be seen in Figure 9a that the temperature distribution near the right mounting port was relatively uniform, and there was no obvious hot spot. No obvious shrinkage cavity was found in the photographs of sections of the produced component. In Figure 9b, a small hot spot area was predicted at the left mounting port, and a shrinkage cavity was also found in the section photograph, but the number of defects was small. This proved that the squeeze casting equipment used in this study and shown in Figure 2 could effectively reduce the generation of defects by forcing supplementary pressure at the locations of the left and right mounting ports of the flywheel housing components using a local loading device.

**Figure 10:**
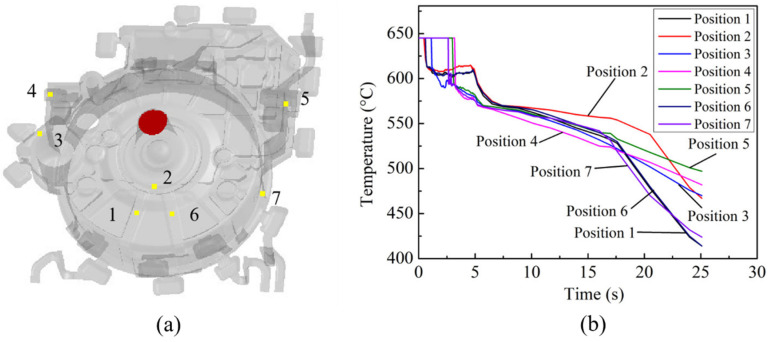
Positions of characteristic points and temperature variation curves with time for test group No. 1: (**a**) characteristic point positions; (**b**) temperature variation curves.

The characteristic points were sampled at seven different positions for the test group No. 1 flywheel housing, as shown in Figure 10a. Positions 1, 2 and 6 were set on the reinforcement rib at the lower surface, position 3 was set next to the larger motor column, positions 4 and 5 were set at the local feeding region and position 7 was set on the cylindrical surface of the flywheel housing. The temperature variation curves with time for the above characteristic points are shown in Figure 10b, where 0~5 s refers to the filling process and 5~25 s refers to the pressure holding process. It can be seen that when the pressure holding process was complete, the temperature at positions 1 and 6 was the lowest, due to the small thickness and the large contact area with the surface of the lower die, leading to faster heat dissipation. The heat dissipation velocity at positions 4 and 5 was less, due to the larger wall thickness, so the temperature was highest at these positions. At position 2, the temperature decreased first but then increased slightly at 4~5 s during the filling process, and the temperature at the end of the pressure holding process was higher than that at nearby positions 1 and 6. This is because position 2 was close to the gate and was first to be filled by the aluminum melt, so the temperature drop occurred first. Then, in the subsequent process of filling the entire mold, the high-temperature molten metal continuously passed through position 2, resulting in a quick recovery in temperature in this region. In addition, because it was closer to the gate, the temperature after the pressure holding process was higher than that at positions 1 and 6 [43]. The results for the temperature–time curves at the characteristic points in Figure 10b were consistent with the analysis of the temperature field distribution at different moments in Figure 6. The solidification rule of the whole flywheel housing component was that the solidification first started in the area away from the gate and then spread to the center of the casting, and the thinner region solidified first, with a higher cooling speed than the thicker regions.

**Figure 11:**
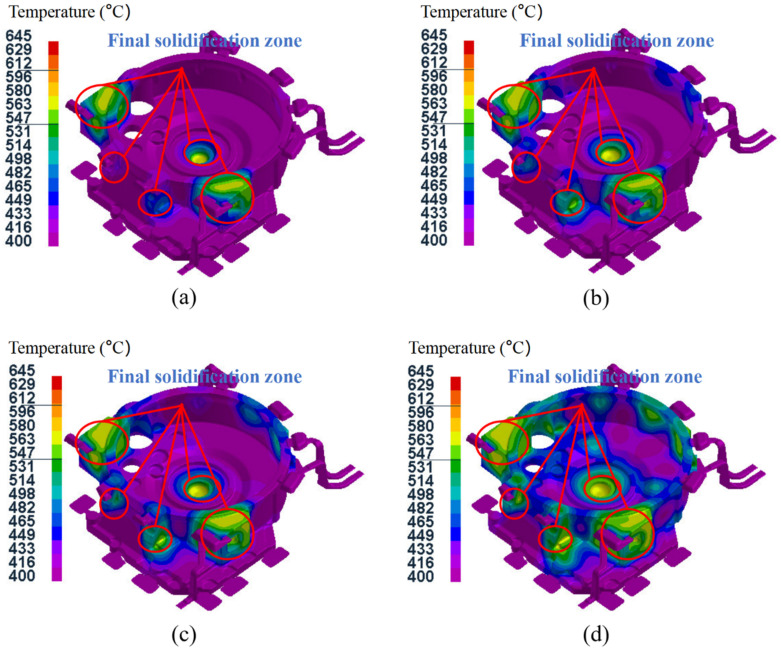
Temperature field distribution after pressure holding process: (**a**) test group No. 9; (**b**) test group No. 10; (**c**) test group No. 11; (**d**) test group No. 12.

Figure 11 shows the temperature field distribution for test groups 9–12 after the pressure holding process. The pressure holding time for these groups was gradually reduced from 35 s to 20 s. It can be seen that the holding time directly affected the temperature field distribution of the aluminum alloy flywheel housing component after the holding time process. The shorter holding time, the higher the average temperature of the flywheel housing component and the more final the solidification zone. At the end of the holding pressure process, in addition to at the left and right mounting ports with large wall thicknesses and the sprue, there was also a small part of the final solidification zone at the convex platform of the cylindrical surface, where it was easy to produce shrinkage cavities and other defects [44]. A local shrinkage compensation effect could be achieved by adding a local loading device to the small convex platform, to inhibit the generation of shrinkage cavities and other defects in this region.

**Figure 12:**
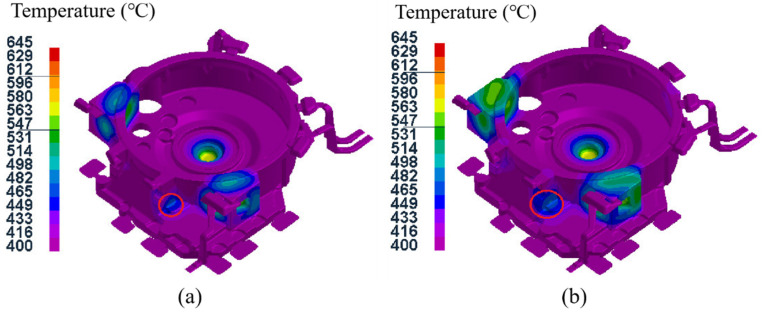
Temperature field distribution of components with and without local loading device at the small convex platform: (**a**) with local loading device; (**b**) without local loading device.

Figure 12 shows the temperature field distribution of the components with and without a local loading device at the small convex platform of the cylindrical surface, under the same process parameters (pouring temperature of 645 °C, mold temperature at level D, holding time of 35 s, specific pressure of 34.00 MPa). It can be seen that the local loading device not only accelerated the cooling at the small convex platform of the cylindrical surface but also reduced the temperature difference between the mounting ports and surrounding areas, which could reduce the shrinkage porosity and other defects.

**Figure 13:**
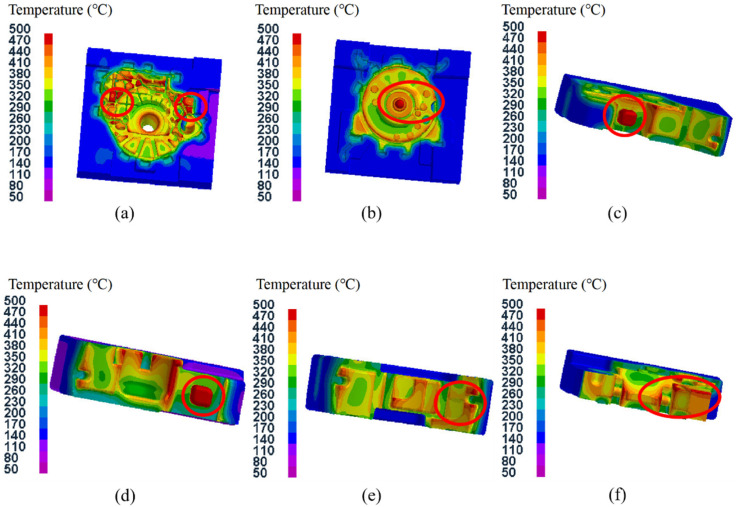
Temperature field distribution of the mold after the packing process for test group No. 1: (**a**) lower die; (**b**) upper die; (**c**) left side die; (**d**) right side die; (**e**) rear side die; (**f**) front side die.

The study analyzed the mold temperature fields of 16 test groups, and the temperature field distribution of each group was roughly the same. Taking test group No. 1 as an example, Figure 13 shows the mold temperature field distribution after the pressure holding process. It can be noted that the mold temperature was unevenly distributed, and the temperature on the surface in contact with the casting increased rapidly, while the temperature in the area away from the casting did not change significantly. The temperatures in regions such as the small convex platform of the lower mold, the diversion cone of the upper mold, the pressure compensation bars of the left and right side molds and the corner of the rear side mold were higher, due to the contact with the casting. Therefore, with repeated use of the mold, the temperature changes in these regions would be relatively large and likely to produce cracks [45,46].

### 3.3. Stress Field Analysis of the Castings

**Figure 14:**
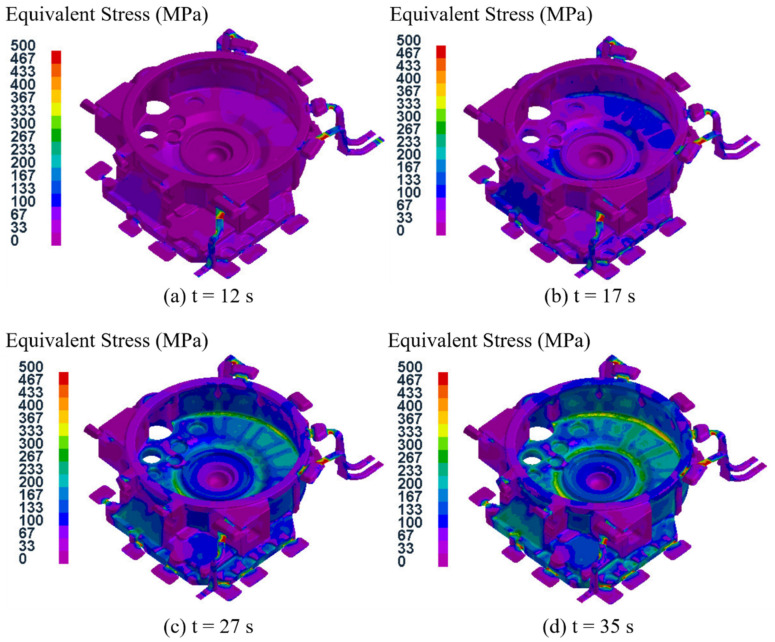
Equivalent stress field distribution at different times during the pressure holding process for test group No. 4.

In this study, the equivalent stress was selected as the object for analyzing the stress of the flywheel housing castings, and the equivalent stress field distribution of test group No. 4 (pouring temperature of 645 °C, mold temperature at level D, pressure holding time of 35 s, specific pressure of 34.00 MPa) in the solidification process is shown in Figure 14. It can be seen that when the pressure holding time reached 12 s, the equivalent stress was relatively larger in the peripheral exhaust groove than that in the nearby overflow groove of the flywheel housing casting, which was due to the larger thickness of the overflow groove relative to the exhaust groove, leading to an uneven contraction of the two regions during the solidification process [47]. When the pressure holding time was 17 s, there was a larger equivalent stress region on the lower surface of the casting. This was because the temperature of the contact surface between the casting and the mold was lower, and the cooling was faster, while the temperature inside the casting was higher and the cooling was slower, and this difference led to an uneven solidification contraction. When the pressure holding time was 27 s, the area with a larger equivalent stress extended from the lower end face to the cylindrical surface, and the equivalent stress was largest at the corner junction between the two parts. This was because the solidification contraction of the lower end face was larger, while the cylindrical surface was firmly fixed on the upper die and the contraction was smaller [48]. When the solidification time was 35 s, the pressure holding process was complete, and the average equivalent stress of the whole flywheel housing was 99.0 MPa.

**Figure 15:**
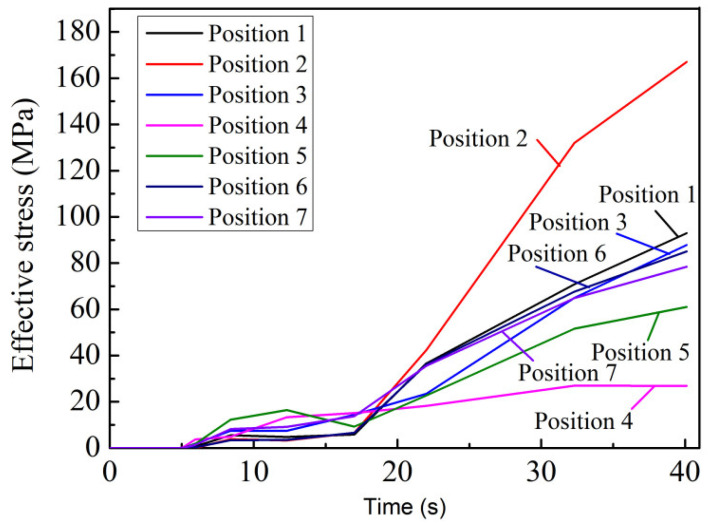
Equivalent stress variation curves with time at each point of test group No. 4.

The characteristic points were sampled at seven different positions in test group No. 4, as shown in Figure 10a, and the equivalent stress variation curves at each characteristic point are shown in Figure 15. In the filling process at 0~5 s, the equivalent stresses at the seven different positions were zero. When the holding pressure process began, the equivalent stresses at the seven different positions all increased gradually as the process proceeded. In the range of 5~17 s, the equivalent stresses at the seven different positions increased slowly and did not exceed 15 MPa. In the range of 17–40 s, the equivalent stresses at the seven different positions increased rapidly. When the time reached 40 s, which was the end of the pressure holding process, the equivalent stress at position 2 was the largest, reaching a maximum of 194.8 MPa, and the equivalent stress at position 4 was the smallest, reaching a maximum of 27.0 MPa. The equivalent stresses at the other five positions were between 60 MPa and 90 MPa, indicating that cracks were more likely to occur at position 2. This is because position 2 was located at the convex platform, which had a small thickness and was closer to the gate. This was the final solidification zone, where the cooling velocity was faster, and the equivalent stress was larger. Position 4 was located at the local pressure position, with large thickness, so here the cooling velocity was slower, and the equivalent stress was lower [49]. The results were consistent with the previous temperature field analysis.

**Figure 16:**
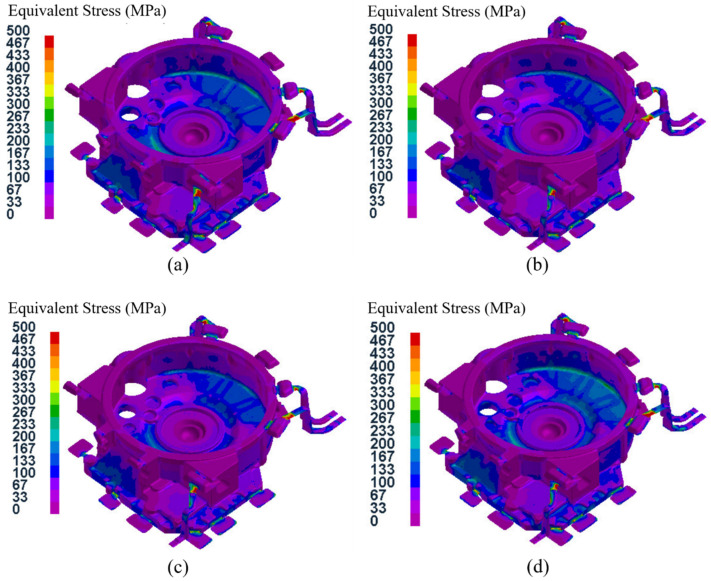
Equivalent stress field distribution of the flywheel housing with pressure holding time of 20 s: (**a**) test group No. 1; (**b**) test group No. 7; (**c**) test group No. 12; (**d**) test group No. 14.

Figure 16 shows the equivalent stress field distributions of the flywheel housing component with a pressure holding time of 20 s in the 16 test groups. It can be seen that the stress field distributions of test groups No. 1, No.7, No. 12 and No. 14 were basically the same, and the areas with high equivalent stress in No. 1 and No. 14 specimens were larger, while those areas in test groups No. 7 and No. 12 were smaller. The maximum average equivalent stress in the four groups was 54.0 MPa in group No. 14, and the minimum average equivalent stress was 43.5 MPa in group No. 12. Among the four test groups, No.14 had the largest specific pressure, and the mold temperature was at level B. The relatively low mold temperature resulted in rapid solidification of the flywheel housing, resulting in a large equivalent stress [50,51], while the mold temperature of group No. 12 was the highest, at level D, which resulted in slow solidification and relatively small average equivalent stress.

**Figure 17:**
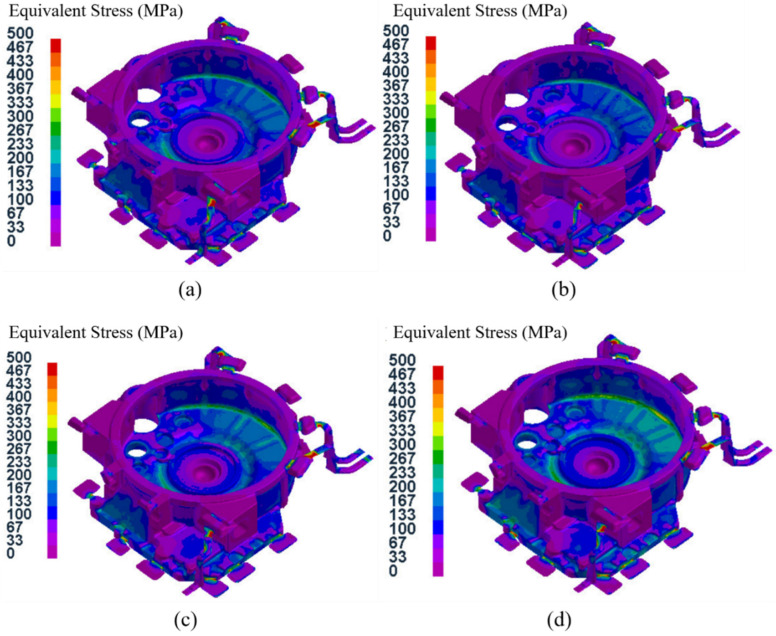
Equivalent stress field distributions of the flywheel housing with pressure holding time of 25 s: (**a**) test group No. 2; (**b**) test group No. 8; (**c**) test group No. 11; (**d**) test group No. 13.

Figure 17 shows the equivalent stress field distributions of the flywheel housing component with a pressure holding time of 25 s for the 16 test groups. Among them, the maximum average equivalent stress of 73.3 MPa was in group No. 13, and the minimum of 56.5 MPa was in group No. 8. Due to the relatively high specific pressure and the lowest mold temperature (level A), the cooling speed of group No. 13 group was relatively fast and the average equivalent stress was the largest. The average equivalent stress in group No. 8, with relatively low specific pressure and the highest mold temperature, was the lowest.

**Figure 18:**
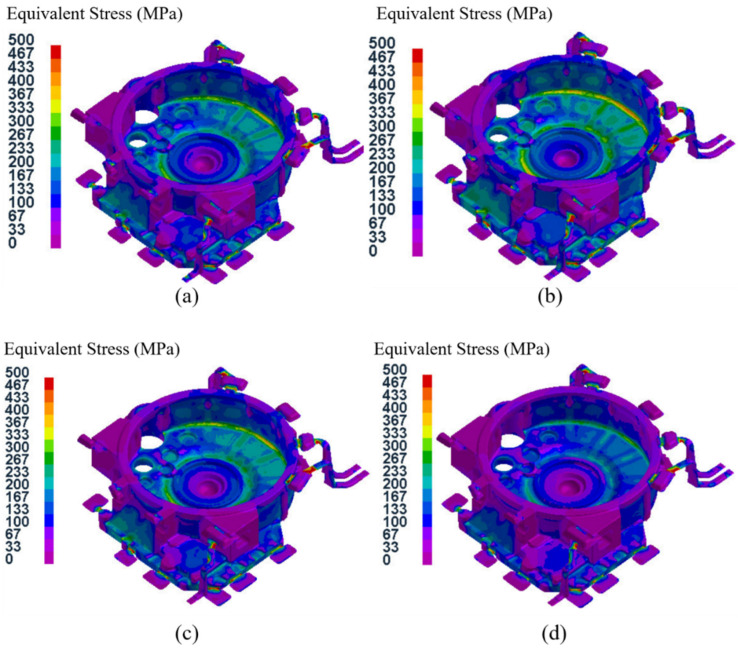
Equivalent stress field distribution of the flywheel housing with pressure holding time of 30 s: (**a**) test group No. 3; (**b**) test group No. 5; (**c**) test group No. 10; (**d**) test group No. 16.

Figure 18 shows the distributions of the equivalent stress fields in the flywheel housing component with a pressure holding time of 30 s in the 16 test groups. Among them, group No. 5 had the highest average equivalent stress of 99.2 MPa, with the largest specific pressure and the lowest mold temperature, and group No. 16 had the lowest average equivalent stress of 67.5 MPa, with the lowest specific pressure and the highest mold temperature.

**Figure 19:**
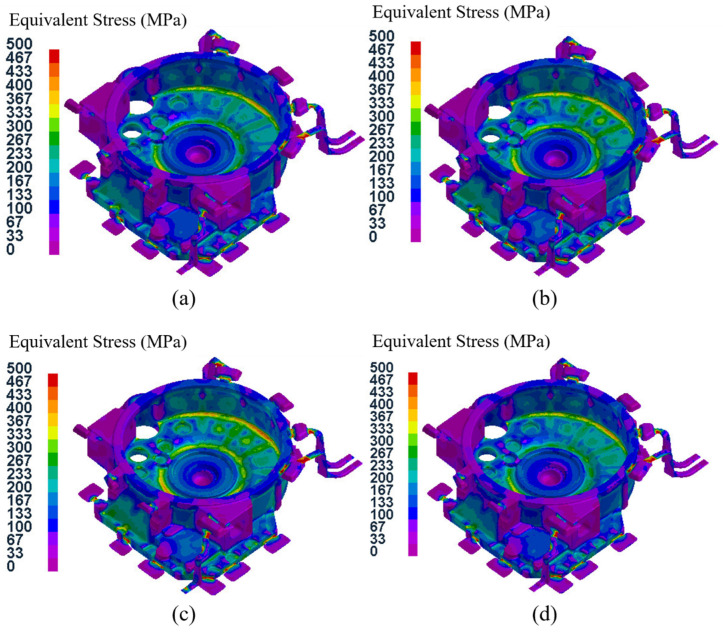
Equivalent stress field distribution of the flywheel housing with pressure holding time of 35 s: (**a**) test group No. 4; (**b**) test group No. 6; (**c**) test group No. 9; (**d**) test group No. 15.

Figure 19 shows the distributions of the equivalent stress field for the flywheel housing with a pressure holding time of 35 s in the 16 test groups. Of the four groups, the mold temperature of group No. 9 was the lowest, and its average equivalent stress was the largest, reaching 111.4 MPa. The mold temperature of test group No. 15 was relatively higher, and the specific pressure was the lowest. Its average equivalent stress was the lowest, at 86.6 MPa.

Based on the above four analyses, the average equivalent stress of the flywheel housing castings increased with an increase in pressure holding time, and the average equivalent stress of the castings with a higher mold temperature was mostly lower than that of castings with a lower mold temperature. This was because a higher mold temperature resulted in the solidification speed of the molten metal being more stable and slower, thus effectively reducing the equivalent stress [52,53]. Among all 16 test groups, the minimum average equivalent stress was found in test group No. 12 (pouring temperature of 655 °C, mold temperature at level D, pressure holding time of 20 s, specific pressure of 28.33 MPa), with an average equivalent stress of 43.5 MPa.

### 3.4. Defects Distribution Analysis of the Castings

**Figure 20:**
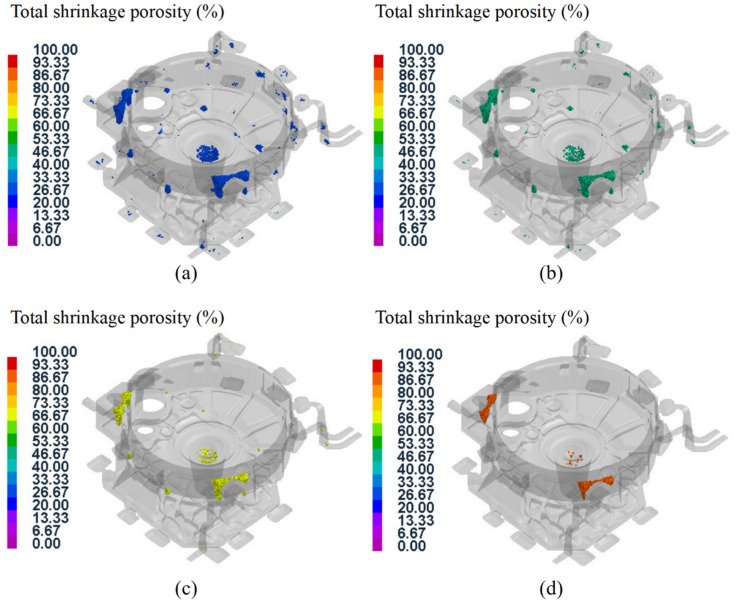
Defect distribution with different porosities for test group No. 1: (**a**) porosity higher than 30%; (**b**) porosity higher than 50%; (**c**) porosity higher than 70%; (**d**) porosity higher than 90%.

Due to the complex and large wall thickness differences in the structure of the flywheel housing component, it is easy to produce hot spots in the squeeze casting process, and this leads to defects such as shrinkage cavities and porosity in many regions. Figure 20 shows the distribution of the shrinkage cavity with different porosities in test group No. 1. It can be seen that the shrinkage cavity was mainly predicted at the left and right mounting ports, the cylindrical convex platform and the peripheral overflow groove, which was consistent with the results of the previous temperature field analysis. In addition, there were also defects at the corner junction with a large thickness variation, due to the uneven cooling contraction in this region [54,55]. The larger the porosity, the smaller the distribution area of defects on the casting. As shown in Figure 20d, for a porosity higher than 90%, defects were less distributed and mainly concentrated at the gate position and the mounting ports.

**Figure 21:**
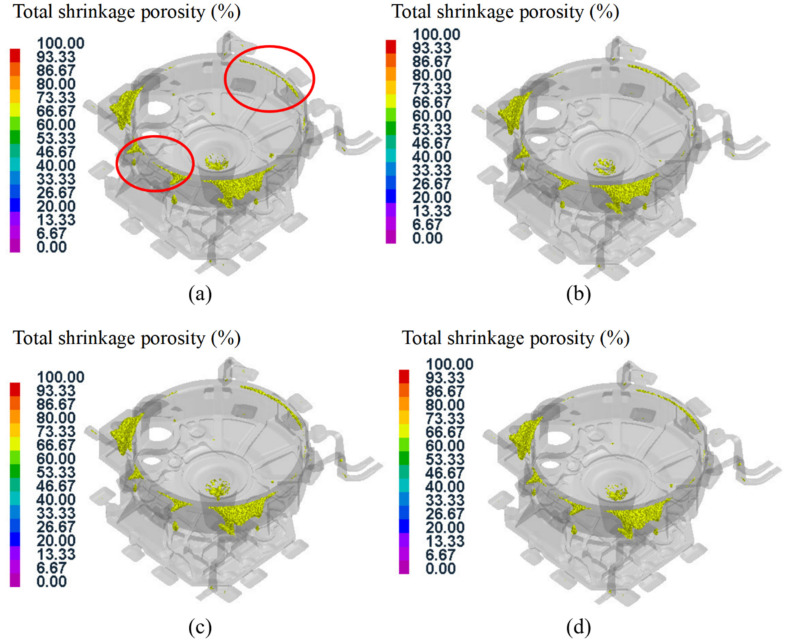
Defect distribution of some test groups: (**a**) test group No. 2; (**b**) test group No. 7; (**c**) test group No. 9; (**d**) test group No. 16.

The shrinkage porosity distributions of the 16 test groups of aluminum alloy flywheel housing components under different parameters were analyzed, and the distribution rule was found to be roughly the same as that of test group No. 1. In addition, some test groups had a small defect region at the peripheral area of the upper end face, as shown in Figure 21. The shrinkage cavity in this region was related to the location away from the gate. The region was finally filled, and because of the convex structure and large thickness difference, the heat dissipation velocity was uneven [56]. If the process parameters such as the mold temperature and pouring temperature were not properly matched, it was easy to produce defects.

Table 7 shows the shrinkage cavity and porosity volume for each group in the simulated tests. By calculating the average defect volume at different levels of a given factor and the range of the same factor, the order of the influence of different factors on the shrinkage cavity volume of the flywheel shell could be determined, and the optimal process parameters could be identified, to minimize the shrinkage cavity volume of the flywheel shell.

Table 8 shows the range analysis results for the effect on defect volume, where K_i_ represents the average value of the defect volume of the flywheel housing at different levels of a given factor and R represents the range under the same factor. It can be seen from the table that a change in the specific pressure caused a change in the defect volume of the flywheel housing of 98.9 cm^3^. This factor had the greatest influence on the defects in the flywheel shell. The change in pouring temperature caused a change in the defect volume of 34.7 cm^3^, and this had the least influence on the defects in the flywheel housing. According to the range analysis results, the effects of the four parameters on the defects in the flywheel housing were in the following order: specific pressure > holding time > mold temperature > pouring temperature. The optimal process parameters for the smallest defect volume in the aluminum alloy flywheel housing were a pouring temperature of 655 °C, mold temperature at level C, pressure holding time of 20 s and specific pressure of 17.00 MPa, giving a volume for the shrinkage cavity and porosity of the aluminum alloy flywheel shell of 100.68 cm^3^.

**Figure 22:**
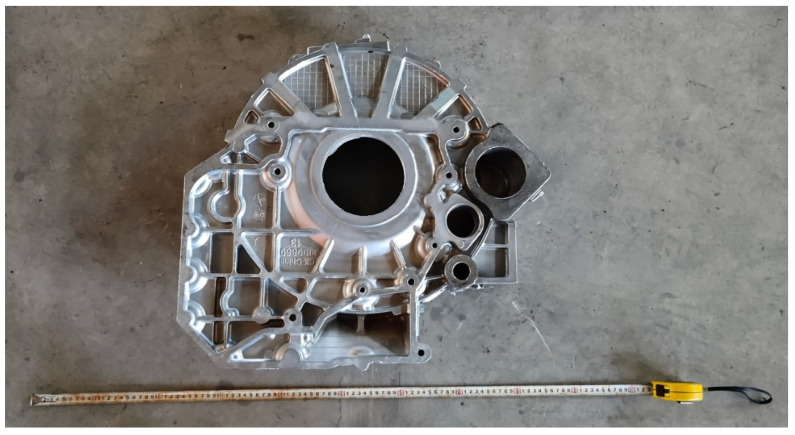
Flywheel housing component produced under the optimal process parameters with the least defect volume, obtained by numerical simulation.

Figure 22 shows the flywheel housing component produced under the optimal process parameters with the least defect volume, obtained by numerical simulation. From the figure, it can be seen that the flywheel shell surface was complete, and there were no obvious macroscopic defects, indicating that a flywheel shell with a good shape can be formed using these experimental parameters.

## 4. Conclusions

1. Flywheel housing castings in all 16 simulation test groups were fully filled, with no insufficiently filled regions. The filling rules were as follows. The molten metal entered the mold cavity from the sprue in the anti-gravity direction and began to fill the lower surface of the flywheel housing first. After 5 s, the filling process was complete, and the semisolid alloy maintained good liquidity with a low solid fraction, which made it possible to exert a certain pressure for forced feeding through the lower injection cylinder during the subsequent solidification process.
2. The solidification rules of the flywheel housing component were as follows. The solidification first occurred in the area away from the gate and then spread to the center of the flywheel housing, and the thinner regions solidified first with faster cooling than the thicker regions. Finally, the solidification was complete at positions including the sprue, left and right mounting ports and cylindrical convex platform, and the final solidification at the sprue position was conducive to reducing defects in the castings. Due to the large wall thickness of the mounting ports and the convex platform on the cylindrical surface, it was easy to produce hot spots, but a local shrinkage compensation effect could be achieved by adding a local loading device.
3. In the temperature field distribution diagram of the mold, it can be seen that the temperature was relatively higher at regions such as the small convex platform of the lower mold, the diversion cone of the upper mold, the pressure compensation bars of the left and right side molds and the edge of the corner of the rear side mold. With repeated use of the mold, it was easy to produce cracks in these regions.
4. The equivalent stresses at the lower end face and the corner junction were larger due to the different solidification speeds. Castings with a longer pressure holding time and a lower mold temperature had a larger average equivalent stress. Among all 16 test groups, the minimum average equivalent stress was found in test group No. 12 (pouring temperature of 655 °C, mold temperature at level D, pressure holding time of 20 s, specific pressure of 28.33 MPa), with an average equivalent stress of 43.5 MPa.
5. The shrinkage cavity was mainly distributed at the left and right mounting ports, the cylindrical convex platform, the peripheral overflow groove and the corner junction with a large thickness variation, which was consistent with the results of the temperature field analysis. There was also a small defect region at the peripheral area of the upper end face in some test groups with unsuitable process parameters. According to the range analysis results, the optimal process parameters for the smallest defect volume in the aluminum alloy flywheel housing were a pouring temperature of 655 °C, mold temperature at level C, a pressure holding time of 20 s and a specific pressure of 17.00 MPa.

## Figures and Tables

**Table 1:** Chemical composition of AlSi9Mg aluminum alloy.

Element	Si	Mg	Mn	Cu	Fe	Cr	Zn	Al
Mass fraction (%)	8.790	0.194	0.246	0.074	0.295	0.030	0.027	Balance

**Table 2:** Preheating temperature levels for each part of mold (°C).

Temperature Level	Lower Mold	Upper Mold	Left, Front and Rear Side Molds	Right Side Mold
Level A	150	160	150	120
Level B	160	170	160	130
Level C	170	180	170	140
Level D	180	190	180	150

**Table 3:** Experimental settings for four specific pressure levels.

Parameter	Level 1	Level 2	Level 3	Level 4
Force of the lower injection cylinder (T)	60	80	100	120
Specific pressure (MPa)	17.00	22.66	28.33	34.00

**Table 4:** Parameters and influence levels of numerical simulation of flywheel housing components formed by squeeze casting.

Parameters Concerned	ID	Influence Level			
		Level 1	Level 2	Level 3	Level 4
Pouring temperature (°C)	I	645	650	655	660
Mold temperature (°C)	II	A	B	C	D
Pressure holding time (s)	IV	20	25	30	35
Specific pressure (MPa)	V	17.00	22.66	28.33	34.00

**Table 5:** Simulation plans for numerical simulation of flywheel housing components formed by squeeze casting.

Test Group	Influence Level					I	II	III	IV	V
	I	II	III	IV	V	Pouring Temperature (°C)	Mold Temperature (°C)	—	Pressure Holding Time (s)	Specific Pressure (MPa)
1	1	1	1	1	1	645	A	—	20	17.00
2	1	2	2	2	2	645	B	—	25	22.66
3	1	3	3	3	3	645	C	—	30	28.33
4	1	4	4	4	4	645	D	—	35	34.00
5	2	1	2	3	4	650	A	—	30	34.00
6	2	2	1	4	3	650	B	—	35	28.33
7	2	3	4	1	2	650	C	—	20	22.66
8	2	4	3	2	1	650	D	—	25	17.00
9	3	1	3	4	2	655	A	—	35	22.66
10	3	2	4	3	1	655	B	—	30	17.00
11	3	3	1	2	4	655	C	—	25	34.00
12	3	4	2	1	3	655	D	—	20	28.33
13	4	1	4	2	3	660	A	—	25	28.33
14	4	2	3	1	4	660	B	—	20	34.00
15	4	3	2	4	1	660	C	—	35	17.00
16	4	4	1	3	2	660	D	—	30	22.66

**Table 6:** Corresponding heat transfer coefficient at different specific pressures.

Parameter	Level 1	Level 2	Level 3	Level 4
Specific pressure (MPa)	17.00	22.66	28.33	34.00
Heat transfer coefficient (W·m^−2^·°C^−1^)	3602.10	4138.67	4676.18	5213.70

**Table 7:** Shrinkage cavity and porosity volume of each simulated test group.

Test Group	Pouring Temperature (°C)	Mold Temperature (°C)	Pressure Holding Time (s)	Specific Pressure (MPa)	Defect Volume (cm^3^)
1	(1) 645	(1) A	(1) 20	(1) 17.00	103.98
2	(1) 645	(2) B	(2) 25	(2) 22.66	236.08
3	(1) 645	(3) C	(3) 30	(3) 28.33	246.98
4	(1) 645	(4) D	(4) 35	(4) 34.00	247.01
5	(2) 650	(1) A	(3) 30	(4) 34.00	247.32
6	(2) 650	(2) B	(4) 35	(3) 28.33	247.73
7	(2) 650	(3) C	(1) 20	(2) 22.66	221.63
8	(2) 650	(4) D	(2) 25	(1) 17.00	251.43
9	(3) 655	(1) A	(4) 35	(2) 22.66	242.21
10	(3) 655	(2) B	(3) 30	(1) 17.00	108.86
11	(3) 655	(3) C	(2) 25	(4) 34.00	243.26
12	(3) 655	(4) D	(1) 20	(3) 28.33	235.03
13	(4) 660	(1) A	(2) 25	(3) 28.33	241.72
14	(4) 660	(2) B	(1) 20	(4) 34.00	236.83
15	(4) 660	(3) C	(4) 35	(1) 17.00	114.42
16	(4) 660	(4) D	(3) 30	(2) 22.66	247.81

**Table 8:** Range analysis of the effects on defect volume.

Factor	Pouring Temperature	Mold Temperature	Pressure Holding Time	Specific Pressure
K_1_	208.512	208.808	199.368	144.673
K_2_	242.027	207.375	243.123	236.933
K_3_	207.340	206.572	212.743	242.865
K_4_	210.195	245.320	212.843	243.605
R	34.687	38.748	43.755	98.932

## Data Availability

The raw/processed data required to reproduce these findings cannot be shared at this time due to technical or time limitations.
